# Postprandial dynamics of splenic volume in healthy volunteers

**DOI:** 10.14814/phy2.14319

**Published:** 2020-01-24

**Authors:** Lydia Garnitschnig, Johannes Weinzirl, Lukas Andrae, Tom Scheffers, Thomas Ostermann, Peter Heusser

**Affiliations:** ^1^ Institute for Integrative Medicine Faculty of Health Witten/Herdecke University Herdecke Germany; ^2^ Department of Internal Medicine Community Hospital Herdecke Herdecke Germany

**Keywords:** digestion, postprandial hyperemia, splanchnic circulation, spleen, splenic rhythm

## Abstract

Throughout the history of medicine, many functions have been attributed to the spleen and numerous researchers have focused on a postulated digestive function. Beginning in 1825, systematic animal studies showed evidence for a postprandial increase in splenic volume (SV) with a peak 30 min to five hours after food intake. Since the introduction of imaging techniques, two studies have been conducted on humans, revealing a decrease in SV 30 to 45 min postprandially. The aim of this study was to examine possible postprandial changes in SV over a period of seven hours. The ethics‐approved, randomized crossover study included 10 healthy volunteers, who received a standardized meal (3,600 kJ) on one study day and fasted on the other. Sonographic measurements were obtained at six measurement points on each day. Thirty minutes after the meal, SV increased significantly by 38.2 ± 51.2 cm^3^ (17.3%; *p* = .04) compared to the baseline measurement and decreased gradually afterward. In males, SV 30 min after the meal was 70.2 ± 21.6 cm^3^ higher (*p* = .002) compared to the fasting condition and 60 min later it was still significantly increased. The apparent SV increase after food intake is discussed in relation to hemodynamic changes in the splanchnic region. It seems plausible that the spleen has a rhythmic and regulative function within the portal system, something which warrants further research and should be taken more into account in nutritional physiology.

## Graphical abstract

In our crossover study, 10 healthy test subjects were randomly given either a substantial meal (3,600 kJ) or remained fasting. Splenic volume was systematically followed up using sonographic video measurements at five measurement points over seven hours. Splenic volume increased significantly with a peak 30 min after food intake. We discuss these findings in relation to hemodynamic changes in the portal system and postulate that the spleen functions as a rhythmic compensatory organ within the splanchnic circulation, something which should be considered to a greater degree in nutritional physiology.

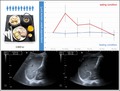

## 1 INTRODUCTION

The spleen is considered to be a neglected organ and its function appears to have been occult for a long time (Bowdler, 2002). Before being classified as a lymphoid organ, it was considered as one of the four major organs in humoralism. A correlation particularly with digestion has been discussed since antiquity (Weinzirl, Scheffers, Garnitschnig, Andrae, & Heusser, 2020). Plato described the spleen as a sponge to absorb “impurities” in the region of the liver that result from certain disorders. Filled with these excretions, the spleen swells and festers, but shrinks again when the body is purged. Aristotle, who was one of the first who speculated on the possible digestive functions of the spleen, thought that it assists the stomach by draining “superabundant moisture” from it (Paraskevas, Koutsouflianiotis, Nitsa, Demesticha, & Skandalakis, 2016). Galen also postulated an exchange of “juices” or blood between the spleen and stomach. These ancient physiological concepts persisted in essence until the Renaissance. Anatomical text books described changes in the splenic volume related to digestive processes up until the late 19th century (Huxley, 1869).

The first systematic studies on postprandial volume changes in the spleen took place in the 19th century. Between 1825 and 1941 several authors observed a postprandial increase in splenic volume in animal experiments, usually post‐mortem examinations (see Table 1)*.* The maximum volume was measured in a time range from shortly after food intake to 15 hr after, with a peak five hours postprandially. Henry Gray, who himself studied the weight of the spleen in rabbits, summarized the state of research in his first edition of “Anatomy: Descriptive and Surgical”: “The size of the spleen is increased during and after digestion” (Gray, 1858).

**Table 1:** Overview of studies between 1825 and 2000 on postprandial changes in the spleen (h = hours, min = minutes, na = not available, PME = post‐mortem examination, pp = postprandial, SV = splenic volume)

Author(s)	Experimental objects/subjects	Testing method	Results
(Leuret & Lassaigne, 1825)	Dogs, cats, rabbits, guinea pigs, and other mammals (*n* = na)	PME	Increase in SV at the beginning of the intestinal phase
(Czermak, 1829)	Rabbits (*n* = 10)	PME	SV bigger in rabbits that ate shortly before the examination
(Dobson, 1830)	Dogs (*n* = 8)	PME	Increase in SV starting 3 hr pp with a maximum 5 hr pp and a considerable decrease in SV 12 hr pp
(Schwager‐Bardeleben, 1841)	Dogs (*n* = 2)	PME	Increase in SV with the beginning of digestion in the stomach
(Landis,^1847^)	Rabbits (*n* = 30)	PME	Maximum increase in SV 5–12 hr pp
(Dittmar, 1850)	Humans (*n* = 11)	Percussion of the spleen	Increase in SV starting 3 hr pp with a maximum 5–6 hr pp
(Stinstra, 1854)	Cats (*n* = 2), dogs (*n* = 8), rabbits (*n* = 3)	PME	Increase in SV at the time of the absorption of the chyle; in dogs: increase in SV starting 3 hr pp with a maximum 5 hr pp; in rabbits: big, tight spleen 5–8 hr pp, small, loose spleen 18 hr pp
(Gray, 1854)	Rabbits (*n* = 30)	PME	Increase in splenic weight with a maximum 10–15 hr pp
(Schönfeld, 1855)	Rabbits (*n* = 6)	PME	Increase in splenic weight with a maximum 5 hr pp
(Hargis & Mann, 1925)	Dogs (*n* = na)	Plethysmography	Protein‐rich food: increase in SV with a maximum 20 min pp, decrease starting 7 hr pp; fat‐rich food: increase in SV with a maximum 24 min pp, decrease starting 9 hr pp; carbohydrate‐rich food: initial decrease then increase in SV with a maximum 45 min pp and return to the baseline volume 4–5 hr pp
(MacKenzie et al., 1941)	Mice (*n* = na)	Transillumination of the exteriorised spleen	Increase in SV after food intake
(Mislin, 1941)	Salmon (*n* = na)	PME	Enlarged spleen during feeding period
(Roshdy et al., 1997)	Humans (*n* = 20)	SPECT	Decrease in SV 30–45 min pp
(Betal et al., 2000)	Humans (*n* = 10)	MRI	Decrease in SV 30–45 min pp

In the modern literature there are two studies which have used imaging techniques to examine the effects of food intake on splenic volume in healthy volunteers. Both showed a decrease in splenic volume 30–45 min after a standard meal. Roshdy, Larsson, Kimiaei, and Jacobsson (1997) found a mean decrease of 3.2% (*p* < .01) in 20 healthy participants using single photon emission computed tomography (SPECT). Betal, Hughes, Whitehouse, and Roberts (2000) described a mean decrease of 6.6% (*p* < .01) in 10 healthy participants using magnetic resonance imaging (MRI). The findings were explained by postprandial dynamics in the splanchnic blood flow. In both studies, measurements were performed only once after food intake. In fact, to date no study has investigated the effects of food intake on splenic volume in healthy volunteers using medical imaging techniques over a period greater than 30 min.

The primary and predefined aim of this exploratory study was to examine postprandial changes in splenic volume over a period of seven hours. Our hypothesis tested in this study was that food intake leads to a sonographically measurable change in the splenic volume over a period of 30 min to seven hours.

## 2 MATERIALS AND METHODS

A within‐subject crossover design was used in this exploratory study. The splenic volume of ten healthy participants was measured by sonography on two days with a one‐week interval. Exclusion criteria were obesity (body mass index (BMI) ≥35 kg/m^2^) (Moriggl, 2009), current use of medication (in particular vasoactive drugs, corticosteroids, contraceptives, anorectics, statins, antacids, and proton‐pump inhibitors), consumption of alcohol more than twice a week (William & Corazza, 2007), consumption of more than three cups of coffee or three energy drinks per day (Boekema, Samsom, van Berge Henegouwen, & Smout, 1999), nicotine dependence (Pandit, Sikora, Muralidhar, Rao, & Sriramarao, 2006), liver disease, diabetes, pregnancy, and lactation period.

Based on the exploratory aim and on the results of the two previous studies (Betal et al., 2000; Roshdy et al., 1997), which had recorded significant changes in splenic volume with 10 and 20 participants, a sample size of 10 participants was chosen. In addition, this number allowed constant examination conditions (examination of all participants within each test day by the same examiner), and the participants could serve as their own control, increasing the internal validity of the design.

In total, 10 healthy participants (five men and five women; mean age of 28 ± 5.7 years; BMI 22.8 ± 4.0 kg/m^2^) participated. They were assigned to two randomized, gender‐matched groups, one of them receiving a standardized meal and 360 ml lime blossom tea on the first day of the study and fasting on the second, the other group fasting on the first day and eating the standardized meal on the second. The balanced meal weighed 700 g and contained about 3,600 kJ (with a protein content of 14%, fats 28%, and carbohydrates 58%).

Six sonographic measurements were performed on each day at standardized time points. The initial measurement was made after overnight fasting (T0). Approximately one hour after the baseline measurement one group received a meal while the other one remained fasting. Measurements took place 30 min (T1), 90 min (T2), three hours (T3), five hours (T4), and seven hours (T5) afterward. All measurements were performed under the same conditions and by the same examiner (experienced in abdominal ultrasonography), who was blinded to group assignment. Conventional ultrasound was used (mid‐frequency curvilinear probe; Aplio 500; Canon Medical Systems Europe). The participants were in supine position with their left arm extended upwards in order to open the intercostal spaces (Benter, Klühs, & Teichgräber, 2011). After locating the splenic hilum in longitudinal section, they were asked to inhale and exhale calmly while video sequences were recorded for 15–30 s. The same procedure was then performed in cross section. The sonographic examinations took 3–5 min in total. During the examination, heavy respiratory maneuvers were avoided, as even short apneas can result in sympathetically mediated contractions of the spleen (Inoue, Nakajima, Mizukami, & Hata, 2013). In general, it was endeavored to run the study in a calm manner in order to reduce sympathetic stress reactions of the spleen (Stewart & McKenzie, 2002). This was reinforced by asking the participants to avoid extreme physical and mental tasks before the baseline measurement, as well as between the points of measurement within the course of the study. All participants used public transportation or their own car to get to the place, where the study was conducted. Before each measurement, the participants rested at least for 10 min in a sitting position.

To ensure the best possible comparability of all measurements from one patient, splenic volume was calculated later with single frames from the video sequences. These frames were selected according to predefined criteria: minimal shadowing, maximum visibility of the margins, and maximum diameter of the spleen. Maximum length (ML) was measured as the maximum diameter in longitudinal section, craniocaudal length (CCL) as the distance between the inferior and superior margins (maximum convexity) in longitudinal section, and width (W) as the maximum diameter between the medial and lateral margin in cross section. The following formula developed by Pilström and evaluated in numerous studies to calculate changes in splenic volume (Engan, Richardson, Lodin‐Sundström, & Schagatay, 2013; Richardson, Lodin, Reimers, & Schagatay, 2008; Schagatay, Richardson, & Lodin‐Sundström, 2012) was used to determine splenic volume:

$$\frac{\text{ML}\times \pi \times \left(W\times \text{CCL}-{\text{CCL}}^{2}\right)}{3}$$

The data collected were analyzed using the statistical package for the social sciences (SPSS Software) and Microsoft Excel. All results were expressed as means ± standard deviation. First, a within condition analysis of variance (ANOVA) was performed to detect changes in the course of time for both the eating and the fasting group. Differences were calculated between the baseline measurement (T0) and possible peaks (T1–T5) using paired sample *t*‐tests. Second, a between condition ANOVA with repeated measures was performed to detect differences between the eating and fasting group. Equality of multiple variance‐covariance matrices was checked through Box's M test (*p* = .172). The homogeneity of error variances was verified using Levene's test (*p* > .05 for all variables). Finally, a univariate sensitivity analysis was carried out to detect possible gender related effects. Results were considered as significant when the *p*‐value was <0.05.

The present study complies with the Declaration of Helsinki and was approved by the Ethics Committee of Witten/Herdecke University. All participants gave their written informed consent before being included. The datasets generated and analyzed during the study are available from the corresponding author on reasonable request.

## 3 RESULTS

All 120 planned measurements were carried out successfully. Splenic volume at time T0 (baseline) showed no significant difference (*p* = .55) between the fasting and eating conditions (see Table 2), with a mean of 223.5 ± 54.1 cm^3^. The stable baseline with comparable initial values for both conditions and both gender groups (see below) confirmed the internal validity of the within‐subject crossover design.

**Table 2:** Mean value (MV), standard deviation (*SD*), minimum (MIN), and maximum (MAX) splenic volume [cm^3^] in absolute terms; *n* = 10 (5 males, 5 females)

Subjects	Measurements	MV	*SD*	MIN	MAX
Under fasting condition (*n* = 10; 5 males, 5 females)	T0 (−1 hr)	226.13	50.87	180.51	362.29
	T1 (+0.5 hr)	223.87	58.02	176.00	384.36
	T2 (+1.5 hr)	225.48	56.97	168.75	374.21
	T3 (+3 hr)	227.22	65.19	169.35	406.37
	T4 (+5 hr)	226.28	60.36	169.95	383.20
	T5 (+7 hr)	222.40	56.30	167.72	374.41
Under eating condition (*n* = 10; 5 males, 5 females)	T0 (−1 hr)	220.86	59.87	169.74	378.11
	T1 (+0.5 hr)	259.07	89.77	91.28	440.71
	T2 (+1.5 hr)	235.63	77.85	99.96	390.40
	T3 (+3 hr)	238.09	55.47	172.84	369.15
	T4 (+5 hr)	227.41	74.12	140.28	370.96
	T5 (+7 hr)	215.39	59.82	140.35	342.01

**Figure 1:**
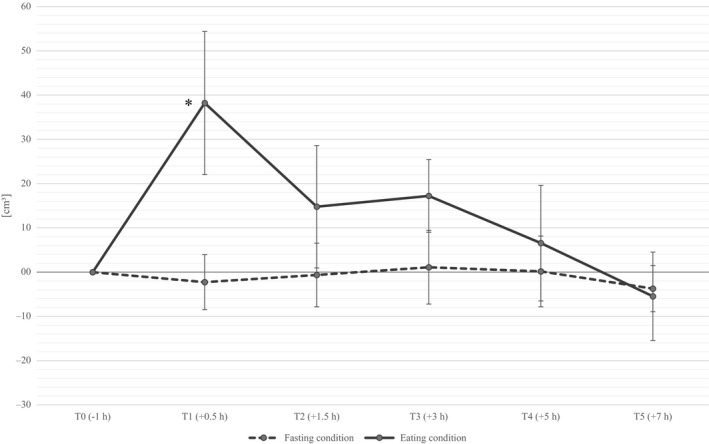
Difference of the mean splenic volume [cm^3^] compared to the baseline (T0) (including standard error) under fasting and eating conditions; *Significant (*p* < .05) in univariate analysis with paired sample *t*‐tests; *n* = 10 (5 males, 5 females)

**Figure 2:**
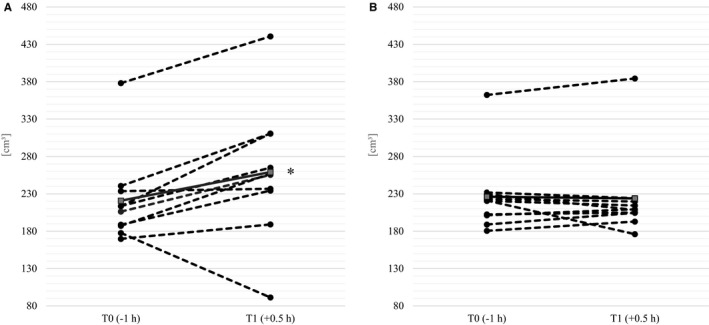
Individual data points (circles) and mean values (squares) of splenic volume [cm^3^] at the baseline (T0) and 90 min afterward (T1) under eating (a) and fasting (b) conditions; *Significant (*p* < .05) in univariate analysis with paired sample *t*‐tests

The within condition ANOVA showed a significant increase of splenic volume (*p* = .03) under the eating condition with a peak 30 min after food intake (T1), while in the fasting condition the splenic volume remained relatively constant over all measurement times (*p* = .9). Thirty minutes after food intake splenic volume increased on average by 38.2 ± 51.2 cm^3^ (17.3%; *p* = .04) compared to the baseline measurement and returned to the initial value after five to seven hours (see Figure 1). Figure 2 shows the individual results in change from time T0 to T1.

The between condition ANOVA was not able to detect a general between group difference for all time points (*p* = .06). However, a significant between group difference of splenic volume at T1 was found, where the mean splenic volume in the eating group was significantly larger than in the non‐eating group (40.5 ± 53.7 cm^3^; *p* = .04).

The above‐mentioned differences were greater in a gender‐based evaluation. While there was no significant difference between men and women in baseline splenic volume (*p* = .10), male splenic volume at T1 and T2 was significantly higher by 70.2 ± 21.6 cm^3^ (*p* = .002) and 32.0 ± 22.8 cm^3^ (*p* = .03), respectively, in comparison to the fasting condition. No significant differences between the fasting and eating conditions were recorded for women. This result is due to the record of a female test subject in whom the splenic volume showed a marked decrease (−86.4 cm^3^ at time T1). If this record is omitted, then splenic volume in women increases by an average of 14.8% (T1).

## 4 DISCUSSION

The aim of the present study was to examine possible postprandial changes in splenic volume. To the best of our knowledge, this is the first sonographic study to report a significant increase in splenic volume after eating in healthy test subjects. Splenic volume seems to increase with a peak 30 min after food intake. At this time point the splenic volume in the eating group was significantly larger as compared to baseline (38.2 ± 51.2 cm^3^; *p* = .04) as well as compared to the fasting condition (40.5 ± 53.7 cm^3^; *p* = .04). The increase in splenic volume tended to remain until five hours after food intake (T4). The baseline was restored seven hours after eating (T5).

The generalizability of these results might be limited by our sample size, investigation method and environmental factors. Concerning the sample (*n* = 10) the stable baseline measurements with comparable initial values for both conditions confirmed the internal validity of our within‐subject design. Nevertheless, the chosen sample might not be representative. Larger study designs are needed to reduce a possible sampling error.

Concerning our investigation method, sonography is the method of choice for spleen examination (Arishenkoff et al., 2015; Klinischer, 2002; Lamb et al., 2002; Nuernberg, Ignee, & Dietrich, 2006). Precise determination of splenic volume is, in principle, possible per ultrasound (Benter et al., 2011; Lamb et al., 2002; Nuernberg et al., 2006; Yetter, Acosta, Olson, & Blundell, 2003). However, the value of the measurements depends essentially on the skill and experience of the examiner. We tried to ensure the best comparability as all measurements were performed by the same well trained and highly experienced examiner within a standardized procedure. Nevertheless, further studies with alternative imaging techniques would be welcome.

Regarding environmental factors, our crossover design on two examination days carries the risk of fluctuations of day‐dependent factors. On the second investigation day, outside temperature (21°C to 36°C) was up to 12.8°C higher than on the first day (8.8°C to 23.2°C). Room temperature, humidity, and other environmental factors were constant in the study room itself. If needed, the participants had free access to drink still water between the measurements. Special care was taken to avoid fluid intake for at least 30 min before the next examination, since half of the consumed fluids should have passed the stomach within 10 to 20 min after intake. However, fluid intake and excretion were not recorded in detail. In general, as mentioned above, the subjects were asked to abstain from strenuous activities and the use of digital media in the time between the measurements. All subjects adhered to these guidelines and there were no abnormal activities.

It is interesting to note that, using SPECT and MRI, Betal et al. (2000) and Roshdy et al. (1997) found a significant *decrease* in splenic volume 30–45 min after food intake, which would at first appear to be in contrast to our findings. Methodically it is not clear whether and for how long breathing maneuvers were undertaken in these studies, which could have influenced the results. The spleen is sensitive to sympathetic stress reactions (Stewart & McKenzie, 2002). Even short apneas of only a few seconds lead to occasional contractions of the spleen in order to provide the circulation with pooled blood (Baković et al., 2003; Inoue et al., 2013). Future studies should pay particular attention to the possibility of breathing or stress‐related contractions of the spleen.

The aim of this study was to examine spleen volume changes only and not to find possible physiological explanations. Nevertheless we suggest that the volume increase 30 min after eating seems to have a direct connection to the splanchnic circulation, as postprandial splanchnic blood flow increases by up to 200% at this time, depending on the quantity and composition of the food (Matheson, Wilson, & Garrison, 2000). Also, the contradiction between an increase of splenic volume (result from this study) as well as a decrease (Betal et al., 2000; Roshdy et al., 1997) 30 min after food intake might be explained by a closer examination of the splanchnic circulation. The spleen fulfills two different functions there, depending on the pressure conditions in the portal system, a topic recently reviewed (Weinzirl, Garnitschnig, Scheffers, Andrae, & Heusser, 2020) as follows: The healthy spleen displays an interesting basic rhythm, which was first discovered in animal experiments and confirmed in human beings using the method of splenoportography. The spleen undergoes continuous contractions and dilatations lasting for a period of about one minute (Wannagat, 1967). This must be distinguished from the sympathetically innervated single contractions which lead to an expulsion of blood from the spleen, such as in apneic diving (Baković et al., 2003), sports activities (Stewart & McKenzie, 2002), or ischemic stress reactions (Liu et al., 2015; Shephard, 2016). A distinction must also be made from the congestive dilatations which occur for example in chronic liver diseases (Iwakiri, 2014).

Hemodynamic studies from the late 20th century show that an increase in portal pressure after eating leads to stronger rhythmic responses of the spleen, a reduction in its arterial blood flow due to a venovasomotoric reaction and therefore a relief of the portal system (Gorjajew, 1932; Lutz, Henrich, Peiper, & Bauereisen, 1969; Schneider, 1967). This might result in a decrease in splenic volume, as observed by Betal et al. (2000) and Roshdy et al. (1997). However, if the postprandial portal flow increases further, the spleen cannot cope with the increased pressure, the rhythm ceases and a compensatory pressure equalization takes place with swelling of the organ (Ewerbeck, 1949; Streicher, 1961). This function of a “hepatolineal blood pendulum” (Henschen & Reissinger, 1928) has also been described as the “regulatory principle of the portal system” (Streicher, 1961), the spleen being a “pressure reservoir” (Ewerbeck, 1949), an “auxiliary motor” (Gorjajew, 1932), a “pump” (Guillery, 1938), or a “windkessel function” in analogy to the systemic circulation (Henschen & Reissinger, 1928).

A key factor in the degree of postprandial hyperemia and therefore the change from the initial decrease in size of the spleen to the engorged organ is the quantity and composition of the food. Solid, well‐balanced, calorie‐rich food leads to a greater blood flow than liquid, low‐calorie food (Matheson et al., 2000). While the test subjects in Roshdy et al. (1997) received 1,600 kJ (decrease in splenic volume of 3.2%) and in Betal et al. (2000) 2,460 kJ (decrease in splenic volume of 6.6%), the participants in our study were given a significantly more substantial meal of 3,600 kJ (increase of 17.3%). We therefore interpret the increase in volume of the spleen as resulting from a greater splanchnic hyperemia which leads to a pressure equalization with enlargement of the spleen, ceasing after about 3 hr.

In this regard, mention can be made of the interesting hypothesis of Steiner and Kolisko who studied the regulatory functions of the spleen, particularly in relation to the dietary rhythm (Kolisko, 1922). Apart from meal size, they postulated that *irregular food intake* would put greater strain on the digestion and make demands on the compensatory function of the spleen. They suggested that taking regularly‐timed, smaller, low‐fat, and low‐carbohydrate meals with a corresponding reduced hyperemia in the splanchnic region could relieve the hemodynamic strain on the liver and spleen (Weinzirl, Scheffers, Garnitschnig, & Heusser, 2018). This might have a practical implication in conditions like portal hypertension, for example, in chronic liver disease, where dietary measures might also be adjusted to the dynamics of the splanchnic area.

Our study confirms the older literature which reported an enlargement of the spleen immediately postprandially, based on post mortem examinations in animal experiments (Czermak, 1829; Hargis & Mann, 1925; Leuret & Lassaigne, 1825; MacKenzie, Whipple, & Wintersteiner, 1941; Mislin, 1941; Schwager‐Bardeleben, 1841; Stinstra, 1854). We could not confirm the observations of studies which reported a later maximum in splenic volume after 3 to 5 hr (Dittmar, 1850; Dobson, 1830; Gray, 1854; Landis, 1847; Schönfeld, 1855; Stinstra, 1854). It should be noted that the experiments on animals are only applicable to humans to a limited degree, as the structure and function of the spleen—especially the storage spleen in cats and dogs—differs from the spleen in human beings (Tischendorf, 1969).

In our study, there appears to be an interesting difference between men (distinct increase in volume of 28.5% after 30 min) and women (no significant difference from baseline). This result could be attributed to the record of a female test subject in whom the splenic volume showed a marked decrease (−86.4 cm^3^) 30 min postprandially. We interpret this response as a stress‐related single contraction, particularly as the test subject admitted in the study protocol that she had a migraine starting. But even without this record, the postprandial response was greater in the spleens of male test subjects than in those of females. An additional explanation might be the different body weight in women (mean BMI in male test subjects 25.4 kg/m^2^, mean BMI in female test subjects 20.1 kg/m^2^; *p* < 0.01). We would recommend to adjust meal size per kilogram bodyweight in future studies.

Our study provides evidence of a significant postprandial increase in splenic volume which is compatible with the hypothesis that the spleen has a rhythmic compensatory function within the splanchnic circulation. This suggests that apart from the liver, the spleen might play an important regulatory role in nutritional metabolism, something which should be considered to a greater degree in nutritional physiology. Further systematic studies with larger sample sizes and alternative imaging techniques would be required to confirm the postprandial responses of the spleen within the splanchnic area. Follow‐up studies should pay attention to the quantity, composition and intake time of the meals as well as to the spleen's sensitivity to sympathetic stress reactions.

## CONFLICT OF INTEREST

The authors declare that they have no conflict of interest. The study was not influenced by the foundations that provided financial support.

## AUTHOR CONTRIBUTIONS

LG and JW developed the concept and design of the research which was reviewed by PH. LA collected the ultrasound data. LG and TO analyzed the data. LG prepared the figures. LG and JW interpreted the results of the measurements and wrote the final manuscript. The manuscript was reviewed, edited, and revised by TS and PH.
